# The Regulation of NF-κB Subunits by Phosphorylation

**DOI:** 10.3390/cells5010012

**Published:** 2016-03-18

**Authors:** Frank Christian, Emma L. Smith, Ruaidhrí J. Carmody

**Affiliations:** Centre for Immunobiology, Institute of Infection, Immunity and Inflammation, College of Medicine, Veterinary and Life Sciences, University of Glasgow, Glasgow G12 8TA, UK; frank.christian@glasgow.ac.uk (F.C.); e.smith.4@research.gla.ac.uk (E.L.S.)

**Keywords:** NF-κB, phosphorylation, kinase, transcription factor

## Abstract

The NF-κB transcription factor is the master regulator of the inflammatory response and is essential for the homeostasis of the immune system. NF-κB regulates the transcription of genes that control inflammation, immune cell development, cell cycle, proliferation, and cell death. The fundamental role that NF-κB plays in key physiological processes makes it an important factor in determining health and disease. The importance of NF-κB in tissue homeostasis and immunity has frustrated therapeutic approaches aimed at inhibiting NF-κB activation. However, significant research efforts have revealed the crucial contribution of NF-κB phosphorylation to controlling NF-κB directed transactivation. Importantly, NF-κB phosphorylation controls transcription in a gene-specific manner, offering new opportunities to selectively target NF-κB for therapeutic benefit. This review will focus on the phosphorylation of the NF-κB subunits and the impact on NF-κB function.

## 1. Introduction

### 1.1. NF-κB

The NF-κB transcription factor plays a fundamental role in a number of key physiological processes. Although most studied for its central role in the development, homeostasis and activation of the immune system, NF-κB is also important in the muscular and skeletal systems, and is an important regulator of epithelial homeostasis [1]. At the cellular level NF-κB plays important roles in regulating cell cycle, proliferation and cell death and has been identified as a key factor in aging [2]. These physiological functions of NF-κB are accompanied by equally important roles in disease where NF-κB activity is often found to be dysregulated. Thus, NF-κB play a critical pathological role in inflammatory disease including auto immunity and atherosclerosis, as well as in cancer and neurodegeneration. The therapeutic targeting of NF-κB is an important research goal but efforts to date have been stymied by the importance of NF-κB in normal tissue homeostasis and immunity [3]. However, the key to circumventing such problems may lie in the complexity of the regulatory processes that control NF-κB function, in particular phosphorylation.

NF-κB is of course a family of related transcription factors; RelA/p65, c-Rel, RelB, p50 and p52 that may hetero- and homo-dimerise to form at least 12 different identified dimers. All NF-κB subunits contain a conserved Rel Homology Domain (RHD) that facilitates dimerisation and DNA binding. The RelA/p65, c-Rel and RelB subunits also contain a transactivation domain which is responsible for the transcriptional activity of dimers containing these subunits [4]. The p50 and p52 subunits do not contain a TAD and in the homodimeric form mostly act as transcriptional repressors. The p50 and p52 subunits also share the distinction that they are both generated by the proteolytic processing of a larger precursor protein, p105 and p100, respectively.

NF-κB is activated by a diverse range of stimuli, from immunoreceptors such as Toll-like receptors and antigen receptors, to cytokine and growth factor receptors, as well as physical, oxidative and genotoxic stress (www.nf-kb.org [5]). Phosphorylation plays a critical role in the activation of NF-κB downstream of all these stimuli. In resting cells, NF-κB is sequestered in the cytoplasm through direct interaction with a member of the IκB family of inhibitor proteins such as IκBα. An initiating signal leads to the activation of the IKK complex which contains two IκB kinases, IKKα and IKKβ. Phosphorylation of IκBα by the IKK complex triggers its recognition by an E3 ligase complex containing β-TrCP which leads to its polyubiquitination and subsequent degradation by the proteasome. The liberated NF-κB dimer then translocates to the nucleus where it recognises and binds specific DNA sequences termed κB sites. Although a consensus κB site has been identified (GGGRNNYYCC; R is purine, Y is pyrimidine, and N is any base) [4] there are clear dimer specific preferences for specific κB sequences and recent studies have identified a number of κB sequences with low similarity to the consensus [6]. A second evolutionary conserved pathway towards the activation of NF-κB has also been identified and is referred to as the non-canonical or alternative pathway. This pathway is activated by a relatively small number of TNF receptor superfamily members, including BAFFR, CD40, lymphotoxin β receptor, and RANK, and plays a key role in lymphoid organogenesis [7]. The non-canonical pathway leads to the activation of NF-κB dimers composed of the RelB and p52 subunits and requires the IKKα- and NIK- dependent proteasomal processing of p100 to p52. 

Genes regulated by NF-κB dimers are numerous and unsurprisingly include cytokines, chemokines, immunoreceptors, and regulators of proliferation and apoptosis. The functional nature of such NF-κB target genes underlies the harmful consequences of dysregulated NF-κB and the necessity of tight control of NF-κB activity. The primary means of limiting NF-κB activity is through a negative feedback loop requiring NF-κB induced IκBα expression. Newly synthesised IκBα enters the nucleus, binds NF-κB and translocates it to the cytoplasm, thereby terminating NF-κB directed transcription. More recently the ubiquitination and proteasomal degradation of DNA bound NF-κB subunits has been identified as a major limiting factor of NF-κB mediated transcription independent of IκBα [8]. Over each of the above aspects of NF-κB activity is an additional layer of complexity imposed by the post-translational modification of NF-κB subunits and their regulators. The modification of NF-κB subunits by ubiquitination, acetylation, methylation, nitrosylation and glycosylation has been reported to varying extents but it is the phosphorylation of NF-κB that has received the most attention. 

### 1.2. How Phosphorylation Regulates NF-κB Activity and Function

While there are many post-translational modifications (PTMs) involved in the regulation of the IKK complex and IκB proteins, other important modifications involve the NF-κB proteins themselves [1]. In general terms, the phosphorylation of the NF-κB subunits has a profound effect on the function of NF-κB. As we will discuss below, site-specific phosphorylation of NF-κB subunits controls interactions with other factors, and the stability, degradation and transcription activity of NF-κB dimers. Importantly, it is also becoming apparent that certain phosphorylation events contribute to the selective regulation of NF-κB transcriptional activity in a gene specific manner. It is clear that phosphorylation of NF-κB subunits may either enhance or downregulate the transcription of target genes, or to modulate transcriptional responses, rather than acting as a simple on/off switch. A detailed understanding of such gene specific regulatory processes may hold the key to selective modulation of NF-κB activity for therapeutic benefit.

The phosphorylation events regulating gene transcription activity can be a consequence of signalling from upstream components of the NF-κB pathway, such as IKKα and IKKβ, or from factors that form part of other signalling pathways, thus providing nodes of crosstalk within the wider cellular signalling network. For clarity, the UniProt entries for human RelA/p65 (Q04206), c-Rel (Q04864), p105/50 (P19838), p100 (Q00653) and RelB (Q01201) have been used as reference sequences to assign residue numbers and phospho-residues are numbered accordingly.

## 2. Phosphorylation of the NF-κB Subunits

### 2.1. Processing of p105 and p100

**Figure 1:**
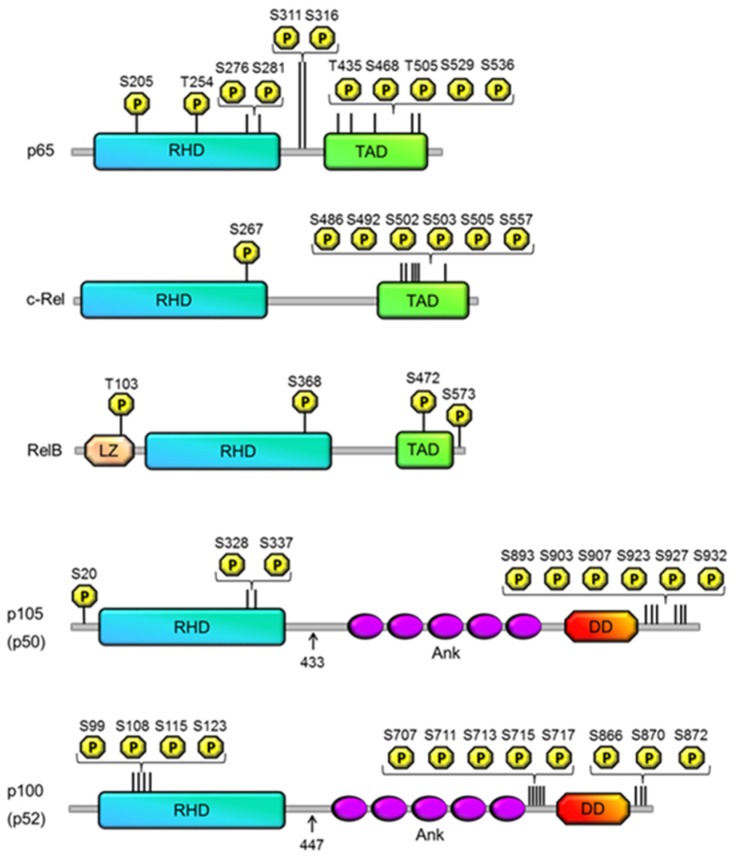
Phosphorylation of the NF-κB subunits. Shown are schematic diagrams of the five members of the NF-κB family: p65 (RelA), RelB, c-Rel, p100 (p52) and p105 (p50). Arrows indicate the C-terminal residues of p50 and p52, which are generated following p105 and p52 processing, respectively. The principal structural motifs for each subunit are indicated, which include the REL homology domain (RHD); transactivation domain (TAD); ankyrin repreat domain (Ank); death domain (DD) and leucine zipper domain (LZ). Phosphorylations that have been shown to regulate NF-κB activity are mapped to each subunit indicating their location relative to each structural motif. The numbering of amino acids corresponds to the human protein sequence.

Both p105 and p100 are synthesized as large precursor proteins, which are partially processed by the proteasome to produce NF-κB subunits p50 and p52, respectively. Phosphorylation plays a central role in the regulation of p100 and p105 processing by the proteasome (Figure 1, Table 1). Limited proteolysis of p105 and p100 is an example of a rare case in which the proteasome does not completely destroy its target, but only partially degrades it [9]. Both precursors also contain C-terminal ankyrin repeats, which confer IκB-like function by sequestering interacting NF-κB subunits in the cytoplasm [2]. Although there are structural similarities between these two precursor proteins, p105 and p100 are subjected to PTMs in distinct pathways, resulting in different functional outcomes.

### 2.2. p105

The p50 subunit is generated through the limited proteasomal processing of the p105 precursor protein, however the precise molecular mechanisms involved remain largely unknown. p105 processing to p50 occurs both constitutively and inducibly and requires a glycine-rich region (amino acids 376–404) which acts as a stop signal for the proteasome [10]. In addition, p105 may be inducibly degraded without the generation of p50. Constitutive processing of p105 may occur co-translationally [11] and post-translationally [12]. Signal induced processing requires site-specific phosphorylation of the C-terminal region of p105 [13,14,15,16,17]. Initial studies identified the phosphorylation of S893 and S907 residues by an unknown kinase as important for the proteasomal processing of p105 [13]. However it is not clear whether p50 is generated as a result of p105 phosphorylation at these sites. Phosphorylation of p105 at S927 and S932 by IKKβ generates a high affinity binding site for βTrCP, the receptor for an SCF-type E3 ubiquitin ligase [15,17] which triggers the ubiquitination of p105 at multiple lysine residues [18]. There are conflicting reports as to the contribution of each of these IKKβ phosphorylation sites to p105 degradation, which may be cell type specific [14,15,16,17]. There are also conflicting reports as to the outcome of IKKβ-mediated phosphorylation of p105 which may lead to complete degradation of p105 or to limited processing to generate p50 [10,15]. Indeed, some studies have revealed two distinct outcomes of IKKβ mediated phosphorylation of p105; (i) stimulation of p105 degradation which is SCF-βTrCP dependent, and (ii) stimulation of p105 processing which is SCF-βTrCP independent. Thus IKKβ phosphorylation may regulate both limited and complete proteolysis *via* two different mechanisms [18]. In addition, p105 is phosphorylated at S903 and S907 by GSK3β [19]. In resting cells, phosphorylation of these residues by GSK3β stabilises p105, and cells lacking GSK3β undergo a higher rate of constitutive p105 processing to p50. S903 and S907 are also required for signal induced p105 processing in response to TNF-α [19]. Although the exact signals that determine whether partial or complete degradation of p105 occurs are presently unclear there is little doubt that phosphorylation is the key determinant.

It should also be noted that p105 has important functions independent of its role as p50 precursor and IκB-like protein. p105 is also an important negative regulator of MAPK activation downstream of a number of receptors including the Toll-like and TNF receptors. A small but distinct pool of p105 is bound to Tpl2 (MAP3K8), an apical kinase of the MAP kinase. p105 serves to stabilise Tpl2 in an inactive form and blocks the activation of the MAP kinase cascade [20,21]. Activation of Tpl2 requires IKKβ phosphorylation of p105 at S927 and S932 and subsequent proteasomal degradation of p105 to liberate the active Tpl2 kinase [21,22]. Free Tpl2 phosphorylates and activates MEK1/2 and triggers a cascade of MAP kinase activity including ERK1/2 [21,22].

### 2.3. p50

To date, the majority of the p105/p50 phosphorylation sites identified are located in the C-terminal region of p105, are regulated by cytoplasmic kinases and as described above, control the processing of p105 by the proteasome. Phosphorylation of the p50 subunit is much less well understood and to date only three p50 phosphorylation sites have been studied in detail; S337, S20 and S328. The phosphorylation of p50 at S337 is critical for DNA binding. S337 lies in a Protein kinase A (PKA) consensus site and PKA has been demonstrated to phosphorylate p50 at S337 *in vitro* and *in vivo* [23,24]. Substitution of serines S65 and S342 with alanine also abolish p50 DNA binding but there is as of yet no demonstration that these sites are phosphorylated. In addition, phosphorylation of human p50 at S20 by DNA-dependent protein kinase (DNA-PK) is critical for expression of VCAM-1 in response to TNFα [25]. However, this serine is not conserved in mouse p50 and so it appears that the regulation of p50 function by phosphorylation at this site is species specific.

The phosphorylation of serine 328 by Chk1 is triggered by DNA damage and regulates the interaction of p50 with specific NF-κB binding elements based on the κB-site nucleotide sequence [26,27]. S328 phosphorylation reduces the affinity of p50 for κB-sites with a cytosine, but not an adenine, at the -1 position. The differential interaction between phospho-p50 and specific κB sequences regulates the transcriptional response following DNA damage by inhibiting the expression of anti-apoptotic genes. As a consequence, p50 S328 phosphorylation promotes DNA damage induced cytotoxicity [28]. In genes with multiple κB-sites, the presence of a single κB-site with a cytosine base at the -1 position is sufficient to inhibit NF-κB-dependent activity. The studies of p50 S328 phosphorylation provide the strongest evidence to date that NF-κB phosphorylation can selectively regulate NF-κB transcriptional activity by modulating DNA binding in a sequence specific manner. The inhibition of gene specific transcription by p50 S328 phosphorylation presumably occurs through altered DNA binding of p50 containing heterodimers (e.g., p50/p65 dimers). Given that p50 has been shown to be an important negative regulator of inflammatory gene expression [29,30], S328 phosphorylation of p50 homodimers may also have an important, and distinct, impact on transcriptional outcomes.

In addition to the above phosphorylation sites of p50, an additional serine residue of p50, S340, has been recently shown to be important for the formation of p50 homodimers [31]. The mutation of S340 to alanine leads to a loss of p50 homodimers and targeted S340A knock-in mice recapitulate the *nfkb1^−/−^* phenotype in a neutrophil driven hepatocellular carcinoma model. Although there is no direct evidence to date that S340 is phosphorylated, the current studies raise the intriguing possibility that p50 dimer formation may be regulated by phosphorylation.

### 2.4. p100 and p52

Similar to p105, the processing of p100 to p52 is regulated by phosphorylation of p100. Although constitutive processing of p100 to p52 does occur [32,37], the majority of p100 appears to be processed in a signal dependent manner. The inducible phosphorylation of p100 is essential for signal induced processing to form p52. p100 processing is activated through the non-canonical pathway, and requires the activation of NIK and IKKα. S866 and S870 of p100 form a binding site for NIK and are essential for NIK dependent recruitment of IKKα to p100 [32]. Once bound to p100 NIK phosphorylates and activates IKKα [38,39]. Although S866 and S870 are phosphorylated in a NIK and IKKα-dependent manner [34], IKKα does not phosphorylate these sites and the kinase responsible remains unclear. Nonetheless, these residues are essential for subsequent p100 ubiquitination and proteasomal processing [33,34]. Once recruited to p100 by NIK, IKKα phosphorylates p100 at S99, S108, S115, S123 and S872, which is required for p100 processing [32]. These phosphorylation events lead to recruitment of βTrCP [33,34,40] resulting in K48-linked ubiquitination of p100 on lysine 856 [41]. In addition, S713 S715 and S717 of p100 are also phosphorylated by an as of yet unidentified kinase [36]. Phosphorylation at these sites contributes to, but is not essential for, p100 processing [33]. More recently, phosphorylation of S707 and S711 by GSK3β has been shown to promote complete proteasomal degradation of p100 involving the E3 ligase Fbw7 [35]. These studies suggest that a pool of p100 is constitutively phosphorylated by GSK3β at S707 and S711, triggering the Fbw7 mediated ubiquitination and proteasomal degradation of p100 which controls the levels of p100 available for the non-canonical pathway.

To date the study of the phosphorylation of p100 in the RHD has focused on the impact of p100 processing. The impact of phosphorylation at S99, S108 and S115 on the function of the p52 subunit has not be explored and additional studies are required to further explore the potential contribution of p52 phosphorylation to the regulation of gene transcription.

### 2.5. RelB

As with p52, the importance of RelB in the non-canonical rather than the classical NF-κB pathway has likely reduced the focus on its regulation by phosphorylation. RelB contains a TAD, cannot form homodimers, and appears to preferentially bind p100/p52 [42]. RelB requires p100 binding for stability [42], and S368 of RelB (human S390) is important for dimerisation with other NF-κB subunits including p100 [43]. Indeed wild type RelB but not a S368A mutant increases the half-life of p100 and reduces the formation of p52 generation. Thus RelB inhibits p100 processing which may be regulated in a signal dependent manner. It should be noted however that the phosphorylation of RelB at S368 has not yet been experimentally demonstrated.

There is strong experimental evidence for the phosphorylation of RelB at threonine 84 (human T103) and S552. Phosphorylation of RelB at these sites is required for the signal dependent proteasomal degradation of RelB induced by T cell receptor activation [44]. RelB degradation is likely to have an important impact on the NF-κB mediated transcriptional response in activated T cells. GSK-3β has been identified as the kinase responsible for phosphorylating RelB at S552, which further highlights an important role for this kinase in the regulation of NF-κB activity [45]. In addition to controlling RelB stability, phosphorylation of RelB also regulates association with IκBα and recruitment to gene promoters. Phosphorylation of RelB at S472 by IKKα and IKKβ inhibits its interaction with IκBα and promotes the expression of a number of genes, including MMP3, which in turn promote TNFα induced cell migration (Table 2) [46].

### 2.6. p65

p65 is the NF-κB subunit that has received the most attention in studies of NF-κB phosphorylation so far, and the bulk of the published information is focussed on the two best-understood phosphorylation targets within the subunit, S276 and S536 (Table 3). In addition to these, nine other phosphorylation sites have been identified. Four of the sites (S205, T254, S276 and S281) are found within the N-terminal RHD, two (S311 and S316) in the linker region C terminal to the RHD, and five phospho-sites (T435, S468, T505, S529 and S536) are located in the C-terminal TAD. The phosphorylation of p65 induces a conformational change, which impacts p65 ubiquitination and stability, as well as protein protein interactions [54].

#### 2.6.1. Phosphorylation of the Rel Homology Domain

##### S276

S276 of p65 is best characterised as a Protein Kinase A (PKA) phosphorylation site. In resting cells the PKA catalytic subunit (PKA-C) is bound in an inactive state to cytosolic IκBα: p65 complexes. Following IKK complex activation and the degradation of IκBα, active PKA-C is liberated and phosphorylates p65 at S276 in a cAMP-independent process [55,56]. The A-kinase interacting protein 1 (AKIP1) promotes both the interaction of p65 with PKA-C and its phosphorylation at S276 [57]. S276 phosphorylation triggers a conformational change in p65 that promotes its interaction with CBP/p300 and thereby increases p65 transcriptional activity [58]. Another node for the regulation of the cytosolic PKA-NF-κB complex exists through cross-talk of the NF-κB and glucocorticoid receptor (GR) pathways [59]. The repression of GR activity by p65 requires PKA phosphorylation of S276 while GR-mediated inhibition of NF-κB activity is also PKA dependent. Thus the cross-repression of NF-κB and GR activity is regulated by PKA associated signalling.

The S276 phosphorylation of p65 and subsequent interaction with CBP/p300 is inhibited by the small GTPase κB-Ras. GDP-bound κB-Ras supresses S276 phosphorylation and decreases the interaction of p65 with CBP/p300 leading to an inhibition of p65 transcriptional activity [60]. S276 phosphorylation appears to selectively promote the expression of a subset of NF-κB regulated genes through the recruitment of cyclin dependent kinase 9/cyclin T1 complexes to target promoters [61]. This gene specific effect of S276 phosphorylation on NF-κB target genes is also seen in embryonic fibroblasts derived from S276A knock-in mice. However, S276A mice die at different embryonic days due to variegated developmental abnormalities [61]. These abnormalities are due to epigenetic changes resulting from the recruitment of histone deacetylases (HDACs) by non-phosphorylated p65 to chromatin. The HDAC recruitment to sites associated with DNA bound S276A p65 leads to the repression of genes located in the proximity of NF-κB target genes and does not appear to be directly related to p65 transcriptional activity [62]. S276 phosphorylation is also important in the p65 dependent epigenetic repression of the tumour suppressor BRMS1 gene [63]. In this case, phosphorylation of S276 is required for the interaction of p65 with DNA (cytosine-5)-methyltransferase 1 (DNMT-1), which leads to the p65 mediated chromatin loading of DMNT-1 at the BRMS1 locus and subsequent DNA methylation and transcriptional silencing. Thus, phosphorylation at S276 may both enhance and repress transcription through different mechanisms in a gene specific manner.

In addition to PKA, p65 S276 phosphorylation is also mediated by a number of other kinases. Phosphorylation of S276 in dendritic cells (DC) is induced following activation of DC-SIGN, a C-type lectin pathogen recognition receptor (PRR) involved in detecting pathogens, such as mycobacteria. DC-SIGN induced S276 phosphorylation is mediated by the Raf-1 kinase and appears to require previous activation of NF-κB through TLR signalling [64]. The phosphorylation of S276 via this pathway is exploited by HIV-1 to productively replicate in dendritic cells, as phosphorylated p65 recruits pTEF-b, containing cyclin T1 and CDK9, to the HIV-1 long terminal repeat (LTR) to promote transcriptional elongation [65]. S276 can also be phosphorylated by mitogen- and stress-activated kinase (MSK)1/2. As opposed to the PKA phosphorylation of p65 in the cytosol, MSK1/2 mediated phosphorylation occurs in the nucleus [66]. This MSK1/2-dependent phosphorylation is activated by a number of stimuli including IL-1α, TNFα, and respiratory syncytial virus (RSV) [67,68], and it has been shown to promote SCF and IL-8 expression during inflammation [69]. Unlike MSK1, MSK2 has been found to be specifically involved in p65 S276 phosphorylation induced by UV irradiation of cells and requires MSK2 activation by CK2 [70]. RSK p90 was found to phosphorylate p65 at S276 in an ERK-dependent pathway, leading to IL-8 expression by intestinal epithelial cells after activation of proteinase-activated receptors (PAR) [71]. Further adding to the kinase multiplicity for S276, macrophage colony-stimulating factor (M-CSF), which promotes mononuclear phagocyte survival and proliferation, activates NF-kB via PKCα phosphorylation of S276 [72].

A recent study of phosphorylation sites within the p65 RHD and their effect on transcription of a set of 37 target genes found that differential phosphorylation of p65 determines transcriptional specificity [73]. The binding of phosphorylated RNA polymerase II (p-RNAP II) to target gene promoters was impaired for a subgroup of 13 genes in a p65 knockdown murine endothelial cell line reconstituted with p65-S276A. Thus, S276 phosphorylation is involved in p-RNAP II recruitment to promoter sites, in addition to regulating the recruitment of transcriptional co-factors such as p300 and p-TEFb. Phosphorylation at S276 is also important in the regulation of other post-translational modifications of p65. PKA phosphorylation of S276 also leads to p65 acetylation at K310 by promoting interaction with CBP/p300 [74]. Acetylation of p65 at K310 also enhances transcriptional activity. Furthermore, phosphorylation of S276 by the Pim-1 kinase prevents p65 ubiquitination to enhance the half-life of p65 and leads to increased transcriptional activity [75].

#### 2.6.2. Other Rel Homology Domain Phosphorylations

A site directed mutagenesis-based screen for potential phosphorylation sites within the p65 RHD identified S205, S276 and S281 as essential for p65 transcriptional activity. Phosphopeptide analysis of p65 containing mutants in these serines strongly suggested S205 and S281 as phosphorylation sites although direct phosphorylation of these sites has not been demonstrated, nor have putative kinases been identified. However, the available evidence suggests that S205 may play a role in sequential p65 phosphorylation events, with phosphorylation of other residues depending on previous S205 phosphorylation [76]. p50:p65-S205A heterodimers and p65 S205A homodimers were found to have a strongly retarded electrophoretic mobility, pointing to a strong effect of the phosphorylation on protein net charge, with potential implications for protein binding partners. However the DNA binding affinity of p65 is not altered by a S205A mutation. S281 forms a motif together with S276 that is conserved across all NF-κB subunits with the exception of p100/p52 [76]. A S281A mutant exhibits impaired DNA binding and reduced transcriptional activity [73]. A comparative analysis of the transcriptional targets of S205A, S276A and S281A mutants of p65 revealed that each potential phosphorylation site contributes to the expression of a distinct subset of genes. Analysis of the κB sequences of these subgroups of genes revealed that the selective regulation of gene expression by p65 phosphorylation may be related to the 5′ guanine content of the κB site. These data indicate an inverse relationship between the 5′ guanine content and the requirement for p65 phosphorylation to promote gene expression and provide further evidence that NF-κB phosphorylation, κB site sequence, and the regulation of transcriptional activity are closely connected [73].

Phosphorylation of p65 at T254 by an unknown kinase is induced by TNFα, and results in a phosphorylated threonine-proline motif that is recognised by the peptidyl-prolyl isomerase Pin1. Pin1-mediated prolyl isomerisation of p65 leads to reduced IκBα binding, p65 nuclear accumulation, increased p65 stability and enhanced transcription [77,78]. Conversely, the mutation of T254 dramatically reduces the half-life of p65 protein and severely limits p65 transcriptional activity. TNFα also induces the PKCζ mediated phosphorylation of p65 on S311. S311 phosphorylation is required for CBP/p300 interaction with p65 and regulates gene expression without affecting nuclear localisation of p65 [79]. Anergic CD8+ T lymphocytes lack phosphorylation of S311, as well as acetylation of K310 indicating S311 as an important regulator of NF-κB activity in T cells [80]. In dendritic cells, PKCζ phosphorylation of p65 at S311 prevents the binding of histone methyltransferase G9a-like protein (GLP) to relieve the repression of p65 target genes [81,82]. IL-1β induced phosphorylation of p65 at S316 leads to increased transactivation and cell growth [83]. Mutagenesis of S316 revealed its contribution to the expression of a distinct sub group of NF-κB-dependent genes, which may occur independently or cooperatively with other phosphorylation sites. *In vitro* analysis indicates casein kinase I (CKI) as a likely kinase for S316 following stimulation with IL-1β.

#### 2.6.3. Phosphorylation of the Transactivation Domain

##### 2.6.3.1. S536

Phosphorylation at S536 in the transactivation domain (TAD) of p65 leads to enhanced transactivation, through increased CBP/p300 binding and acetylation at K310 of p65 [74]. As with S276, a number of kinases have been identified that phosphorylate S536 which include IKKα, Ribosomal Subunit S6 Kinase 1 (RSK1), IKKβ, IKKε and NF-κB activating kinase (NAK)/TANK-binding kinase 1 (TBK1) [84,85,86,87]. Again, the conditions and consequences of S536 phosphorylation would appear to depend on the physiological context.

IKKβ was identified as a p65 S536 kinase over 15 years ago in studies that revealed a dual role for the IKK complex in the activation and regulation of NF-κB [86,88]. During T cell activation, IKKβ phosphorylates S536 within the cytoplasmic IκBα:p65 complex, an event that requires prior phosphorylation of IκBα by IKKβ [89]. Early studies using *p65*^−/−^ MEFs reconstituted with wild type p65 or a S536A p65 mutant suggested that S536 phosphorylation may inhibit nuclear import of p65 [89]. However, subsequent studies using transgenic mice expressing kinase dead IKKα (*IKK^AA^*) identified an important role for S536 phosphorylation in promoting the proteasomal degradation of p65. This appears to be an important mechanism in limiting NF-κB transcription responses in toll-like receptor activated macrophages. In macrophages, the phosphorylation of p65 at S536 by IKKα increases p65 turnover, thereby reducing NF-κB activity and supporting the resolution of inflammation [85]. Interestingly, in these studies IKKα opposes the activatory role of IKKβ in NF-κB modulation. Thus, it may be possible that the increased levels of nuclear S536A p65 previously observed are also a consequence of enhanced p65 protein stability. It is important to note however that independent studies using *IKKα^−/−^* macrophages did not find any significant differences in S536 phosphorylation or p65 half-life [90]. Additional studies also identified casein kinase 1γ1 (CK1γ1) as a S536 kinase. CK1γ1 phosphorylation of p65 at S536 promotes p65 degradation by an E3 ligase complex composed of Cullin2 and COMMD1 to inhibit RIG-I/TLR signalling [91].

An important part of NF-κB regulation is the negative feedback loop that is created through NF-κB induced expression of IκBα. Newly synthesised IκBα enters the nucleus to bind NF-κB dimers and translocate them to the cytosol. In the presence of continuous stimulation, oscillations in NF-κB cytosolic-nuclear translocation are accompanied by oscillations in S536 phosphorylation which suggests that p65 dephosphorylation occurs in the nucleus [92]. While persistent oscillation was found to be required for transcription of target genes, an increase in IκBα led to a frequency reduction of these oscillations, and thereby to a reduction in transcription. However, the nuclear accumulation of IκBα may inhibit NF-κB activity in a gene-specific manner, leading to the repression of pro-inflammatory cytokines [93]. Nuclear IκBα prevents p65 binding to the promoters of *Tnfα*, *Il1β* and *Il6* but does not inhibit the binding of p65 phosphorylated at S536 to the *Il8* promoter. These studies support previous reports that p65 phosphorylated at S536 is not associated with, or regulated by, IκBα and that S536 phosphorylation may lead to the expression of a distinct set of target genes [94]. Thus, the S536 phosphorylation of p65 may determine the ability of IκBα to inhibit transcription in a gene selective manner.

Other inducers of S536 phosphorylation include respiratory syncytial virus (RSV), which not only triggers S276 phosphorylation of p65 through MSK1/2 (see above) but has also been found to also increase S536 phosphorylation through IKKβ [95] and IKKε [96]. In a separate study it was found that TNFα-induced S536 phosphorylation of p65 occurs primarily to the cytosol, while IKKε translocates to the nucleus upon TNFα stimulation of cells, in a process depending on p65 phosphorylation [97]. In the nucleus IKKε can inducibly associate with promoter regions of several NF-κB target genes and contributes to the selective regulation of a subset of NF-κB target genes. Indeed, the phosphorylation of S536 may represent an alternative route to activation of NF-κB by the tumour suppressor p53. p53 expression leads to the activation of ribosomal S6 kinase 1 (RSK1) which phosphorylates p65 at S536 [98]. Phosphorylated p65 has a lower affinity for IκBα, which results in increased nuclear translocation and accumulation of p65. Similarly, an unusual mechanism of oncogene mediated NF-κB activation involving S536, but following neither the canonical nor noncanonical pathway, has also been identified [99]. Here, the oncogenic de-ubiquinating enzyme TRE17/ubiquitin-specific protease 6 (USP6) induces S536 phosphorylation and nuclear retention of p65. This USP6 dependent activation of NF-κB requires IKKα, IKKβ and NEMO in order for p65 S536 phosphorylation to take place.

In DCs, DC-SCRIPT appears to negatively regulate p65 S536 phosphorylation and subsequent acetylation at K310 to inhibit TLR induced IL-10 production [100]. Of note, both IL-6 and TNFα expression levels are not inhibited by DC-SCRIPT, further supporting a gene specific contribution of S536 to the regulation of transcription. Phosphorylation of p65 at S536 is also negatively regulated by the glycosylation of p65 protein with *O*-linked-*N*-acetylglucosamine (*O*-GlcNAc). A reciprocal relationship exists between glycosylation and phosphorylation on S536 such that increased glycosylation inhibits phosphorylation and subsequent gene transcription [77].

##### 2.6.3.2. T435

Phosphorylation of T435, a site located within the TA2 subdomain of the p65 TAD is induced through TNFα stimulation [101]. A phosphomimetic mutation of T435 leads to gene specific effects including increased transcription of CXC chemokine ligand 2 (Cxcl2). This phosphomimetic mutation results in decreased recruitment of HDAC1 to the Cxcl2 promoter leading to elevated levels of histone acetylation. The kinase for T435 is currently unidentified, although CK2 or polo-like kinase 1 are predicted candidates based on the surrounding amino acid sequence of p65. In contrast, dephosphorylation of T435 by the nuclear protein phosphatase 4 (PP4) following treatment of cells with the chemotherapeutic drug cisplatin leads to increased transcriptional activation by p65 suggesting that the consequences of T435 phosphorylation may be cell or stimulus specific [102].

##### 2.6.3.3. S468

To date three kinases have been identified that phosphorylate S468 of p65. GSK3β constitutively phosphorylates S468 in unstimulated cells to negatively regulate basal NF-κB activity [103]. In contrast, IKKε mediated phosphorylation of S468 leads to enhanced transactivation following co-stimulation of T cells [104]. In activated T cells S468-phosphorylated p65 is localised mainly in the nucleus while p65 phosphorylated at S536 is predominantly localised to the cytosol. IKKε mediated phosphorylation of S468 also appears to protect cells against DNA damage-induced cell death [105]. In contrast to the IKKε- and GSK3β-mediated effects, IKKβ-mediated phosphorylation of S468 following IL-1β stimulation appears to take place in the cytosol where S468 phosphorylated p65 is found in complex with IκBα [106]. In these studies, S468A mutation increased NF-κB reporter activity and selectively enhanced TNFα and IL1β inducible expression of Ccl5 but not Ccl2. The enhanced expression of selective p65 target genes may be explained by the finding that phosphorylation of S468 enhances binding of the histone deacetylase GCN5. GCN5 binding facilitates the recruitment of an E3 ligase complex containing Copper Metabolism Murr1 Domain 1 (COMMD1), cullin2 and SOCS1. The acyltransferase activity of GCN5 does not appear to be required for this interaction. The assembled E3 ligase complex promotes the ubiquitination and subsequent proteasomal degradation of p65, which, intriguingly, appears to occur in a gene specific manner to limit gene transcription [107,108].

##### 2.6.3.4. T505

Phosphorylation of T505 has been identified as a means of crosstalk between DNA damage and the activation of NF-κB. T505 phosphorylation is mediated by Chk1 kinase, which may be activated through the tumour suppressor ARF. Phosphorylation at T505 correlates with apoptotic cell death and the inhibition of NF-κB target gene expression including the anti-apoptotic gene Bcl-xL [105,109]. Phosphorylation of T505 also negatively regulates autophagy, proliferation and cell migration indicating that phosphorylation of T505 is important for p65 tumour suppressor activity [110]. The recent generation of a T505A knockin transgenic mouse provides compelling evidence that this is indeed the case. p65 T505A mice display increased cell proliferation in response to liver injury and enhanced sensitivity to chemically induced hepatocellular carcinoma [111]. These studies are the first to employ phosphorylation site-specific mutation of p65 *in vivo* and provide an unprecedented insight into the regulation of NF-κB by phosphorylation in a physiological setting. 

##### 2.6.3.5. S529

S529 is phosphorylated by CK2 upon IL-1β or TNFα stimulation and results in increased p65 transactivation potential in a gene specific manner [112,113,114]. In U937 lymphomatic monocytes, S529 was found to be dephosphorylated upon LPS treatment, whereas S536 phosphorylation was increased [115].

In addition to the discussed Ser and Thr phosphorylation events, it has been shown that the protein-tyrosine kinase Syk is involved in Tyr phosphorylation of p65 [116]. On stimulation of endothelial cells with thrombin, PKCd is activated. This leads to an increase in Syk actvitiy. The effect is NF-kB activation and intercellular adhesion molecule-1 (ICAM-1) expression. A further report demonstrated that the protein-tyrosine kinase c-Src is crucial in this signalling mechanism [117]. The phosphorylated tyrosine residues however were not identified in these studies. 

### 2.7. c-Rel

The c-Rel subunit of NF-κB has been studied primarily in B and T lymphocytes, macrophages and DCs where it is important in cellular differentiation and immune function. The human *c-rel* gene is a susceptibility locus for autoimmunity, including arthritis and psoriasis, and is frequently altered or expression levels increased in B and T cell cancers [118]. Its regulation through phosphorylation remains largely understudied, although an important role for phosphorylation in the regulation of c-Rel function is evident from the available studies.

The PKA consensus site at S276 of p65 as described above is also conserved in the R

HD of c-Rel at S267, however phosphorylation at this site has not been demonstrated to date. However, PKA-Cβ does interact with c-Rel and PKA-Cβ may phosphorylate c-Rel *in vitro* [47]. The consequences of PKA phosphorylation of c-Rel are not clear but early studies suggest enhanced nuclear localisation for a phospho-mimetic S267D mutant of c-Rel [48]. A study in which two amino acids were inserted in the putative PKA site in mouse c-Rel demonstrated that the mutant protein could no longer form DNA-binding homodimers, but could still form DNA-binding heterodimers with p50. This study provides further evidence that specific NF-κB dimer formation might be directed by phosphorylation [119].

Transactivation by c-Rel is dependent on a number of serines in the transactivation domain. Serines between amino acids 540 and 588 in the C terminal region of the TAD appear important for basal transcriptional activity, while serines between residues 422 and 540 appear important for TNFα- and phorbol myristate acetate (PMA)/ ionomycin-induced transcriptional activity. Within this region amino acids 454 to 473 appear important for TNFα induced responses, while residues 492 to 519 appear important for PMA/ionomycin induced responses [51]. NF-κB-Inducing Kinase (NIK) phosphorylates c-Rel between amino acids 460 and 540 leading to activation of the CD28 responsive element in the *il2* gene in T cells [49,50]. Mutation of c-Rel at S460, S471 and S491/494 abrogates NIK and PKCζ induced transcriptional activity in T cells. 

A study analysing point mutations in B cell lymphoma identified a S525 mutation that reduces TNFα and IKKα induced c-Rel reporter activity. Both IKKα and IKKβ phosphorylate S525 *in vitro* [52]. Mutation of S525 conferred resistance to TNFα-induced apoptosis suggesting that this mutation may contribute to the development of B cell lymphoma in humans [52]. It should be noted however that follow up studies failed to support IKK complex mediated phosphorylation of c-Rel as a mechanism for regulating c-Rel transcriptional activity [53]. IKKε and TBK1 may also phosphorylate c-Rel at the C terminus to promote its nuclear accumulation although the specific sites of phosphorylation remain unidentified [120]. In addition, early experiments indicate that c-Rel is also inducibly phosphorylated at as of yet unidentified tyrosine residues [121,122].

## 3. Concluding Remarks

NF-κB subunit phosphorylation and its impact on transcriptional activity brings obvious complexity to the regulation of this physiologically important family of transcription factors. This complexity is due not only to the number of phosphorylation sites, which is likely to be a significant underestimation, but also to the fact that multiple kinases may phosphorylate a single site. Of course, it cannot be assumed that the phosphorylation of NF-κB subunits results in a uniform species of protein, indeed, there already exists significant evidence that phosphorylation of different sites generates a heterogeneous pool of modified NF-κB [97]. It is perhaps this heterogeneity that provides the basis for the gene specific effects of NF-κB phosphorylation, as well as the apparent cell and context dependent functional consequences of individual phosphorylation events. It is also possible that the interaction of multiple identified (and unidentified) phosphorylation sites ultimately determines the selectivity of the effects on NF-κB transcriptional activity.

It is clear from the available evidence that the sequence of NF-κB binding sites in DNA contributes to the selective effects of certain phosphorylation events. However, it is equally clear that the phosphorylation of NF-κB subunits may also regulate the interaction with other factors which in turn may determine the functional consequences. In any case, it is becoming apparent that the regulation of NF-κB subunits by phosphorylation may hold the key to understanding gene-specific regulatory mechanisms and how these may be exploited to achieve selective therapeutic effects.

In the future, we can expect significant advances in our understanding of the physiological roles of specific phosphorylation sites through the use of transgenic knockin mutant mice. The models generated to date provide compelling evidence for the importance of phosphorylation in the regulation of NF-κB function. Genome editing technology such as the Cas9/CRISPR system will provide invaluable tools for the *in vitro* investigation of specific NF-κB phosphorylation sites of in physiologically relevant cells lines. The future use of proteomics to identify the full repertoire of NF-κB phosphorylation will also be essential to fully understand the complexity and interplay of specific sites of phosphorylation. What is certain is that the central role of NF-κB in human health and disease, and the potential benefit of therapeutically targeting this factor, ensures that progress in this area will continue apace.

## Figures and Tables

**Table 1:** Phosphorylations of the p50, p105 and p100 NF-κB subunits.

Site	Kinase	Effect	Reference
**p50**			
S20	DNA-dependent PKA	DNA binding specificity	[25]
S328	Chk1	DNA binding specificity	[26,27,28]
S337	Protein kinase A	Enhanced binding affinity	[23,24]
**p105**			
S893	Unknown	p105 processing	[13]
S903	GSK3β	Resting state: Stabilization TNF-α induced state: ubiquitination	[19]
S907	GSK3β	Resting state: Stabilization TNF-α induced state: ubiquitination	[13,19]
S923	IKKβ	Ubiquitination	[14,15]
S927	IKKβ	Ubiquitination	[17]
S932	IKKβ	Ubiquitination	[14,17]
**p100**			
S99	IKKα	Ubiquitination	[32]
S108	IKKα	Ubiquitination	[32]
S115	IKKα	Ubiquitination	[32]
S870	Unknown	IKKα recruitment	[32,33,34]
S872	IKKα	Ubiquitination	[32]
S707	GSK3β	Ubiquitination	[35]
S866	Unknown	IKKα recruitment	[32,33,34]
S711	GSK3β	Ubiquitination	[35]
S123	IKKα	Ubiquitination	[32]
S713	unknown	Ubiquitination	[36]
S715	unknown	Ubiquitination	[36]
S717	unknown	Ubiquitination	[36]

**Table 2:** Phosphorylations of the RelB and c-Rel NF-κB subunits.

Site	Kinase	Effect	Reference
**RelB**			
T103	Unknown	Degradation	[44]
S390	Unknown	Dimerization	[43]
S573	GSK-3β	Degradation	[44,45]
S472	IKK complex	DNA binding specificity	[46]
**c-Rel**			
S267	PKA (*in vitro* only)	stimulates transcriptional activity, nuclear localisation	[47,48]
S486, S492, S502, S503, S505	NIK?	TNFα-induced transactivation activity	[49,50]
S503	PKCζ, NIK?	TNFα-induced transactivation activity	[51]
S557	IKKα/β (*in vitro* only)	transactivation activity	[52,53]

**Table 3:** Phosphorylations of the RelA/p65 NF-κB subunit.

Site	Kinase	Effect	Reference
S205	unknown	transactivation	[76]
T254	unknown	prolyl isomerisation, transactivation	[77,78]
S276	PKA-C,	transactivation, K310 acetylation	[55,56,58]
	MSK1,		[66,67,68,69]
	MSK2,		[70]
	Pim-1,		[75]
	RSK p90,		[71]
	PKCα		[72]
S281	unknown	transactivation	[73,76]
S311	PKCζ	K310 acetylation, transactivation	[79,80,81,82]
S316	unknown	transactivation	[83]
T435	unknown (CK2, PLK1?)	transactivation	[101,102]
S468	GSK3β	Inhibition	[103]
	IKKε	transactivation	[104]
	IKKβ	slight inhibition	[106]
T505	Chk1	inhibition	[105,109,110]
S529	CK2	transactivation	[112,113,114,115]
S536	IKKβ,	transactivation, K310 acetylation	[84,86,87,88,95]
	RSK1,		[84]
	IKKα,		[84,85,86,87]
	IKKε,		[84,96]
	NAK/TBK1		[84]

## Abbreviations

The following abbreviations are used in this manuscript:

TAD: Transactivation domain
RHD: Rel homology domain
IKK: Inhibitor of kappaB kinase
MAPK: Mitogen activated protein kinase
PKA: Protein kinase A
NIK: NF-kappaB inducing kinase
TLR: Toll-like receptor
CDK: Cyclin dependnet kinase
CK: Casein kinase
GSK: Glycogen synthase kinase
